# Overexpression of a rice *eIF2*β variant modulates leaf age-dependent resistance to pathogens in *Arabidopsis thaliana*

**DOI:** 10.5511/plantbiotechnology.24.1206a

**Published:** 2025-03-25

**Authors:** Yuki Fukamachi, Yui Yamauchi, Atsushi Ishikawa

**Affiliations:** 1 Department of Bioscience and Biotechnology, Fukui Prefectural University, Fukui 910-1195, Japan

**Keywords:** *Arabidopsis thaliana*, eIF2, host resistance, nonhost resistance, *Pyricularia oryzae*

## Abstract

Plants display diverse resistance responses that are influenced by their age and the timing of pathogen exposure. In *Arabidopsis thaliana* (Arabidopsis), nonhost resistance (NHR) to *Pyricularia oryzae* varies with leaf age and the time of inoculation. While the circadian clock and photoperiod have been linked to the time-dependent regulation of NHR in Arabidopsis, the mechanism underlying leaf age-dependent NHR remains unclear. To identify key players in leaf age-dependent NHR to *P. oryzae* in Arabidopsis, we utilized rice-Full-length cDNA OvereXpressing (FOX) Arabidopsis lines and identified the rice *eIF2*β (eukaryotic translation initiation factor 2 beta subunit) variant (Os03g0333300-2). Overexpression of the rice *eIF2*β variant reduced NHR to *P. oryzae* and modulated host resistance (HR) to *Colletotrichum higginsianum* in Arabidopsis. The effect of Os03g0333300-2 expression on resistance is dependent on leaf age in Arabidopsis. These results suggest that overexpression of the rice *eIF2*β variant Os03g0333300-2 could contribute to defense responses in a leaf age-dependent manner in Arabidopsis. Our findings might suggest the involvement of the rice *eIF2*β variant in eIF2-dependent translation regulation of resistance response to pathogens in plants.

Resistance to pathogens in plants varies significantly with leaf age, position, and the developmental stage of the plant (Develey-Rivière and Galiana 2007; Hu and Yang 2019; Li et al. 2020). The phenomenon of age-associated resistance has been extensively studied in various pathosystems and has been found to be linked with phytohormonal regulation in a pathosystem-dependent manner (Xu et al. 2018). However, our understanding of the molecular mechanisms underlying age-related resistance remains limited.

Rice blast disease, caused by *Pyricularia oryzae* (syn. *Magnaporthe oryzae*), is one of the most devastating fungal diseases affecting rice (Ebbole 2007; Koga 2001). While rice is a host for *P. oryzae*, most other plant species are nonhosts. Nonhost resistance (NHR) is the ability of all genotypes of a plant species to resist all genotypes of a pathogen species. Several rice blast resistance genes have been identified in rice, but the mechanisms underlying NHR to *P. oryzae* in nonhost plants, such as Arabidopsis, are not well understood. Previous studies in Arabidopsis have identified several genes, including *PENETRATION 2* (*PEN2*), *MILDEW RESISTANCE LOCUS O 2* (*MLO2*), and *POWDERY MILDEW RESISTANCE 5* (*PMR5*), that are involved in NHR (Maeda et al. 2009; Nakao et al. 2011). *Colletotrichum* species, such as *Colletotrichum higginsianum*, produce appressoria with melanin in their cell walls and have an infection mechanism similar to *P. oryzae* (Yan et al. 2018). Despite extensive research on the mechanisms of host resistance (HR) to *C. higginsianum* in Arabidopsis, the role of Arabidopsis developmental age in regulating HR to *C. higginsianum* remains unknown.

The Full-length cDNA OvereXpressing (FOX)-hunting system has been developed as an alternative to activation-tagging in Arabidopsis (Ichikawa et al. 2006). In this system, the transcriptomes of full-length cDNAs from another plant species are ectopically expressed under the control of the cauliflower mosaic virus 35S promoter in Arabidopsis (Kondou et al. 2009). This approach has been used to identify genes involved in stress tolerance (Yokotani et al. 2008, 2009, 2013), disease resistance (Dubouzet et al. 2011), and other physiological processes.

In this study, to investigate the key factors involved in leaf age-dependent NHR to *P. oryzae* in Arabidopsis, we developed rice-FOX Arabidopsis *pen2 mol2 pmr5 gl1 NahG* (*pm5gN*) lines. A screening of approximately 1,000 rice-FOX Arabidopsis *pm5gN* lines was conducted to identify candidates with altered penetration rates compared to control *pm5gN* plants. Through this screen, we identified a rice *eIF2*β variant, Os03g0333300-2, which suppresses NHR to *P. oryzae* in Arabidopsis *pm5gN* plants. Furthermore, we found that Os03g0333300-2 expression influences HR to *C. higginsianum* in Arabidopsis in a leaf age-dependent manner.

The plants used in this study included Col-0 (wild-type), *pen2 mol2 pmr5 gl1 NahG* (*pm5gN*) (Shimizu et al. 2021), r1 (original name K19720) (Dubouzet et al. 2011), and #52 (this study), which are all on the Col-0 background. Arabidopsis plants were grown in a growth room under controlled conditions as described previously (Maeda et al. 2023) (Supplementary Materials and Methods).

The *Pyricularia oryzae* isolate Hoku 1 (race 007) was cultured on oatmeal medium, and *Colletotrichum higginsianum* (MAFF305635) and *Colletotrichum nymphaeae* (MAFF240037) were cultured on PDA medium. Conidial suspensions of each pathogen were inoculated onto Arabidopsis leaves as described previously (Maeda et al. 2023). Inoculated plants were maintained in a growth chamber with saturating humidity under short-day conditions at 22°C. Infected leaves were harvested at 72 h post-inoculation (hpi). Each experiment included six leaves from six independent plants per genotype and was repeated in triplicate (Supplementary Materials and Methods).

The Agrobacterium library of rice full-length cDNAs was obtained from RIKEN (Kondou et al. 2009). Arabidopsis *pm5gN* plants were transformed using the Agrobacterium library to generate rice-FOX Arabidopsis *pm5gN* lines. Approximately 1,000 lines were inoculated with *P. oryzae* to identify candidate NHR-related lines with different penetration rates from the control *pm5gN* plants (Supplementary Materials and Methods).

In a previous study, we demonstrated that Arabidopsis *pen2 mol2 pmr5 gl1 NahG* (*pm5gN*) plants have reduced NHR to *P. oryzae* compared to *pen2* plants (Shimizu et al. 2021). To identify genes related to the regulation of NHR to *P. oryzae*, particularly in relation to leaf age and time, we developed and screened our rice-FOX Arabidopsis *pm5gN* lines (Supplementary Materials and Methods). We identified one line, #52, which exhibited decreased NHR to *P. oryzae* compared to control plants (Figure 1). To investigate how NHR is regulated in #52 plants depending on leaf age and time, we conducted experiments in which we inoculated young and old leaves of the plants with *P. oryzae* at 10:00 a.m. (am-inoculation) and 5:00 p.m. (pm-inoculation) as previously described (Yamauchi et al. 2017) (Supplementary Materials and Methods). Our results show that inoculation led to decreased NHR in #52 plants, except in old leaves following pm-inoculation (Figure 1). We also identified the overexpressed gene in the #52 plants, which was found to be a rice cDNA AK072674 (Os03g0333300-2) that encodes the rice eIF2β (eukaryotic translation initiation factor 2 beta subunit) variant (Supplementary Figure S1). The rice *Os03g0333300* gene encodes eIF2β and produces three transcripts. Os03g0333300-2 is an alternative spliced variant of the *Os03g0333300* gene (https://plants.ensembl.org/ (Accessed Jan 29, 2025)) (Supplementary Figure S1) and encodes a truncated polypeptide consisting of 100 amino acids (Supplementary Figures S2, S3). The overexpression of the rice cDNA AK072674 (Os03g0333300-2) in #52 plants was confirmed by reverse transcription-polymerase chain reaction (RT-PCR) (Supplementary Figure S4). These findings indicate that the rice *eIF2*β variant plays a crucial role in decreasing NHR in #52 plants, while not having the same effect on old leaves following pm-inoculation.

**Figure 1:**
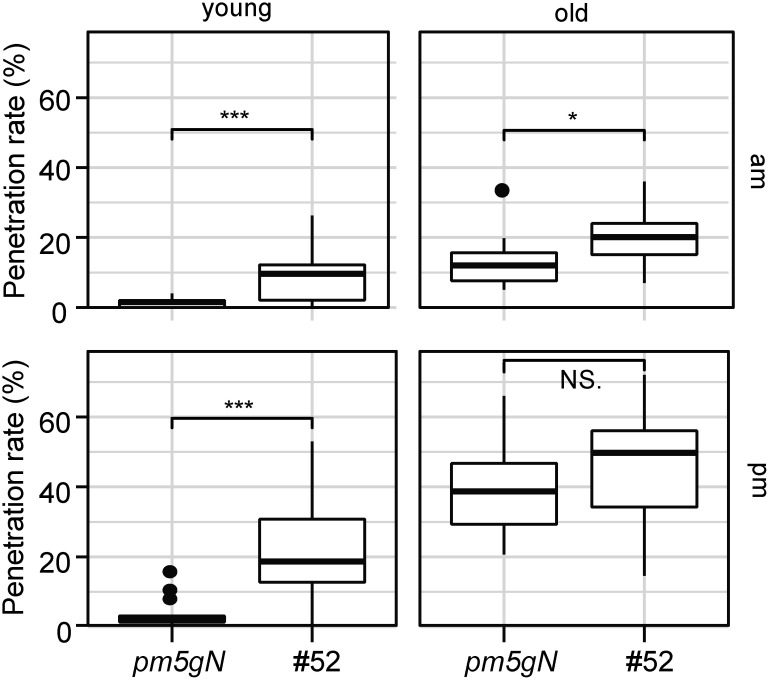
Figure 1. #52 plants show decreased nonhost resistance to *Pyricularia oryzae*. Penetration rate of *P. oryzae* into *pen2 mol2 pmr5 gl1 NahG* (*pm5gN*) and #52 plants at 72 hpi expressed as the percentage of the total number of infection sites. Arabidopsis plants were inoculated at 10:00 a.m. (am) and 5:00 p.m. (pm) on young and old leaves. Values are from three independent experiments, each containing six biological replicates. The student *t* test was used for statistical analysis, NS., not significant; *, *p*<0.05; ***, *p*<0.001.

We inoculated young leaves of Arabidopsis plants with the nonadapted fungal pathogen *C. nymphaeae* and quantified cell penetration. We found that young leaves of *pm5gN* plants showed significantly increased penetration rates compared to wild-type Col-0 plants (Figure 2A). This result suggests that penetration resistance to *C. nymphaeae* was severely compromised in *pm5gN* plants. Then, we tested the effectiveness of rice *eIF2*β variant overexpression against *C. nymphaeae* by examining the #52 plants for NHR. The penetration rate of #52 plants was similar to that of the control *pm5gN* plants (Figure 2A).

**Figure 2:**
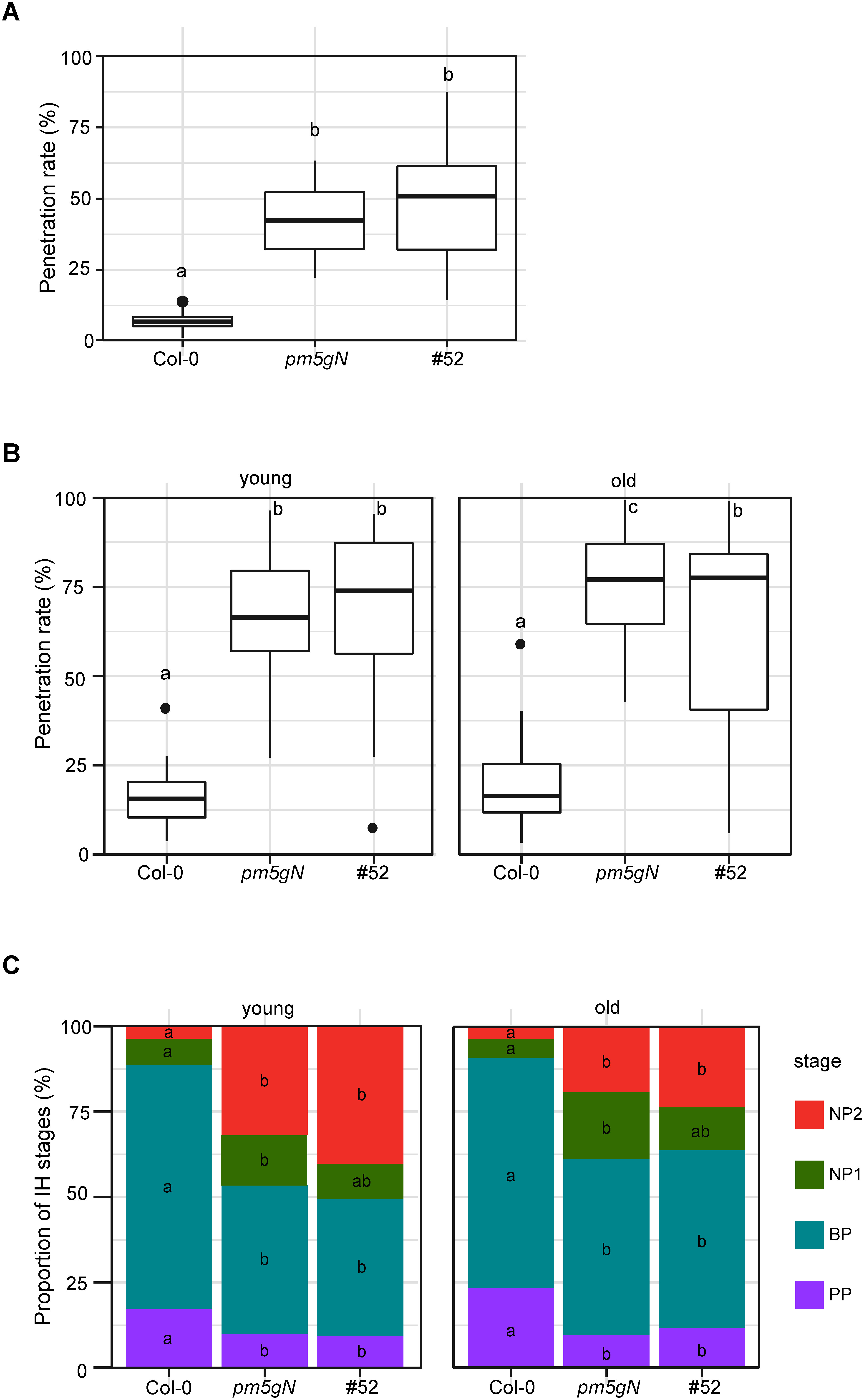
Figure 2. #52 plants show increased host resistance to *Colletotrichum higginsianum*. (A) Penetration resistance to *C. nymphaeae* in #52 plants. Penetration rate of *C. nymphaeae* into Col-0, *pen2 mol2 pmr5 gl1 NahG* (*pm5gN*) and #52 plants at 72 hpi expressed as the percentage of the total number of infection sites. Arabidopsis plants were inoculated at 5:00 p.m. on young leaves. Values are from three independent experiments, each containing six biological replicates. Significantly different statistical groups of genotypes indicated by the analyses of variance (Tukey’s test; *p*<0.05) are shown with lowercase letters. (B) Penetration resistance to *C. higginsianum* in #52 plants. Penetration rate of *C. higginsianum* into Col-0, *pen2 mol2 pmr5 gl1 NahG* (*pm5gN*) and #52 plants at 72 hpi expressed as the percentage of the total number of infection sites. Arabidopsis plants were inoculated at 5:00 p.m. on young and old leaves. Values are from three independent experiments, each containing six biological replicates. Significantly different statistical groups of genotypes indicated by the analyses of variance (Tukey’s test; *p*<0.05) are shown with lowercase letters. (C) Classification of infection hyphae (IH) of *C. higginsianum*. Conidial suspensions of *C. higginsianum* were inoculated on Col-0, *pen2 mol2 pmr5 gl1 NahG* (*pm5gN*) and #52 plants and IH were examined at 72 hpi. The infection process was classified into four stages: penetration phase (PP), biotrophic phase (BP), necrotrophic phase with NH, which are confined within the initially penetrated epidermal cells (NP1), and necrotrophic phase with NH, which spread into the surrounding cells (NP2). Values are expressed as mean from three independent experiments, each containing six biological replicates. Significantly different statistical groups of genotypes indicated by the analyses of variance (Tukey’s test; *p*<0.05) are shown with lowercase letters.

Next, to test if overexpression of the rice *eIF2*β variant gene could contribute to resistance to other pathogens, we investigated its effect against the adapted fungal pathogen *C. higginsianum* in *pm5gN* and #52 plants. We inoculated conidial suspensions of *C. higginsianum* on young and old leaves of Arabidopsis plants. We found that both young and old leaves of *pm5gN* plants exhibited a significant decrease in penetration resistance to *C. higginsianum* compared to wild-type Col-0 plants (Figure 2B). This result suggests that penetration resistance to *C. higginsianum* was severely compromised in *pm5gN* plants. Further, we found that old leaves of #52 plants exhibited a significant increase in penetration resistance to *C. higginsianum* compared to *pm5gN* plants but not young leaves (Figure 2B). These findings suggest that the overexpression of the rice *eIF2*β variant can induce penetration resistance in old leaves of #52 plants against *C. higginsianum*. The host-adapted *C. higginsianum* forms specialized infection structures, such as melanized appressoria, penetrating hyphae, biotrophic hyphae, and necrotrophic hyphae, during its infection process on Arabidopsis. These stages include the penetration phase (PP), biotrophic phase (BP), and necrotrophic phases with necrotic hyphae confined within the initially penetrated epidermal cells (NP1), and necrotrophic phases with necrotic hyphae spreading into the surrounding cells (NP2). We then analyzed the proportion of infection stages of penetrated sporelings in Arabidopsis, as previously described (Maeda et al. 2023) (Supplementary Materials and Methods), and found significant differences between Col-0 and *pm5gN* plants. The proportion of initial penetration (PP) and biotrophic phase (BP) stages in young and old leaves of *pm5gN* plants was significantly decreased compared to Col-0 plants (Figure 2C). Conversely, the proportion of necrotrophic phase stages (NP1 and NP2) in young and old leaves of *pm5gN* plants was significantly increased compared to Col-0 plants (Figure 2C). This result suggests that post-penetration resistance to *C. higginsianum* was severely compromised in *pm5gN* plants. However, #52 plants showed no differences compared to *pm5gN* plants in young and old leaves (Figure 2C). These results indicate that the overexpression of the rice *eIF2*β variant significantly increased penetration resistance but not post-penetration resistance to *C. higginsianum* in old leaves of #52 plants.

To determine whether the phenotypes of the #52 plants (*pm5gN* background) are dependent on the background *pm5gN* mutations, we investigated r1 plants, which overexpress the rice cDNA AK072674 (Os03g0333300-2) in the Col-0 background. The overexpression of the rice cDNA AK072674 (Os03g0333300-2) in r1 plants was confirmed by RT-PCR (Supplementary Figure S4). As *P. oryzae* is almost unable to penetrate epidermal cells of Arabidopsis Col-0 plants (Maeda et al. 2009), we analyzed the interaction between the nonadapted pathogen, *C. nymphaeae*, which can penetrate the epidermal cells of Col-0 plants more efficiently than *P. oryzae* (Irieda and Takano 2021; Maeda et al. 2023), and the r1 plants. We inoculated the plants with conidial suspensions of *C. nymphaeae* on young and old leaves. We found no differences in young and old leaves between Col-0 plants and r1 plants (Figure 3A). Next, we investigated the interaction between the adapted pathogen, *C. higginsianum*, and the r1 plants. We inoculated the plants with conidial suspensions of *C. higginsianum* on young and old leaves. First, we measured the degree of cell penetration. We found no differences in young leaves between Col-0 plants and r1 plants. However, r1 plants showed significantly decreased penetration resistance in old leaves (Figure 3B). Second, to quantify post-penetration resistance to *C. higginsianum*, we investigated the proportion of different infection stages of penetrated sporelings. Our observations showed that old leaves of r1 plants had significantly decreased initial penetration (PP) stages but increased biotrophic phase (BP), and necrotrophic phase stages (NP1) compared to Col-0 plants (Figure 3C). These results indicate that penetration resistance and post-penetration resistance to *C. higginsianum* in r1 plants are severely compromised in old leaves, suggesting that modulation of resistance responses in #52 plants do not require *pm5gN* mutations in the background.

**Figure 3:**
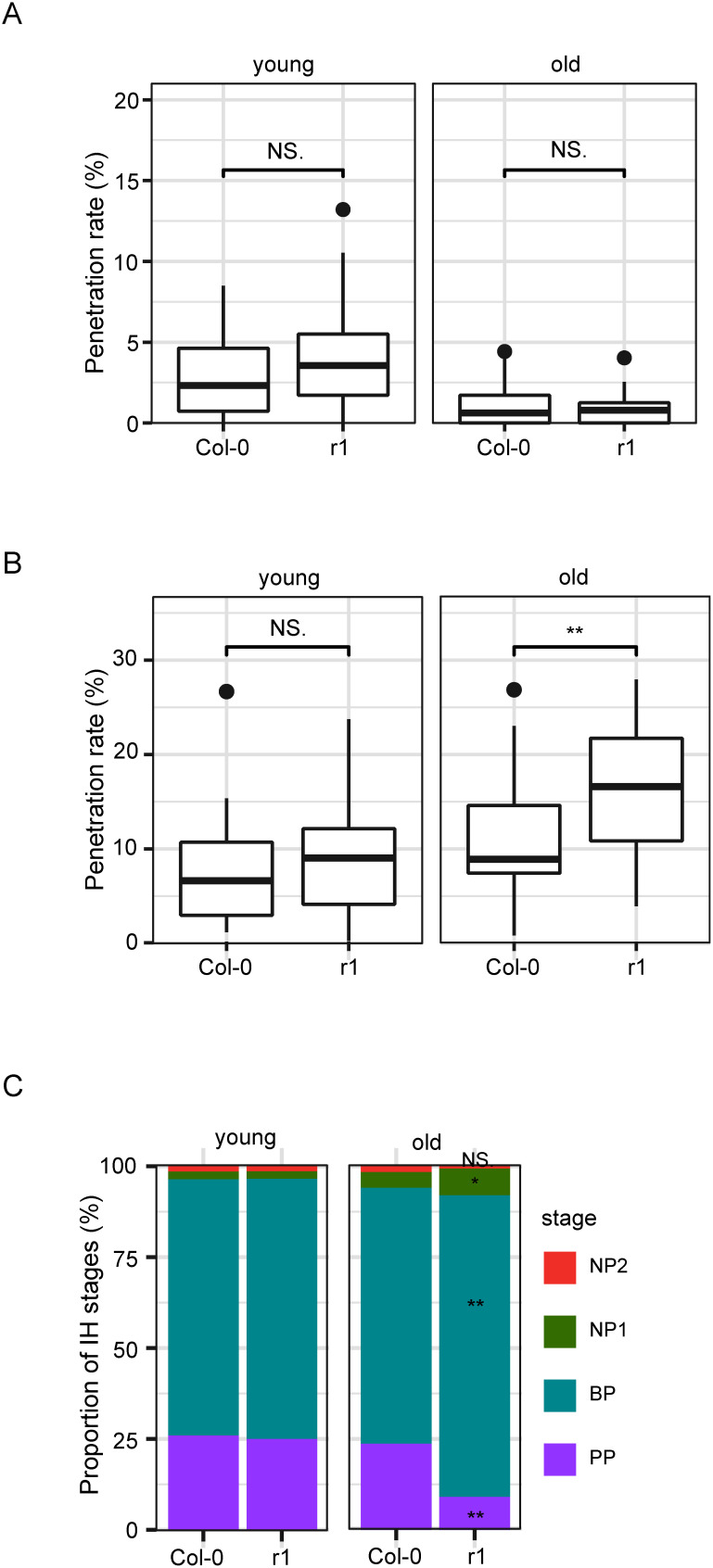
Figure 3. r1 plants show decreased host resistance to *Colletotrichum higginsianum*. (A) Penetration resistance to *C. nymphaeae* in r1 plants. Penetration rate of *C. nymphaeae* into Col-0 and r1 plants at 72 hpi expressed as the percentage of the total number of infection sites. Arabidopsis plants were inoculated at 5:00 p.m. on young leaves. Values are from three independent experiments, each containing six biological replicates. The student *t* test was used for statistical analysis, NS., not significant. (B) Penetration resistance to *C. higginsianum* in r1 plants. Penetration rate of *C. higginsianum* into Col-0 and r1 plants at 72 hpi expressed as the percentage of the total number of infection sites. Arabidopsis plants were inoculated at 5:00 p.m. on young and old leaves. Values are from three independent experiments, each containing six biological replicates. The student *t* test was used for statistical analysis, NS., not significant; **, *p*<0.01. (C) Classification of infection hyphae (IH) of *C. higginsianum*. Conidial suspensions of *C. higginsianum* were inoculated on Col-0 and r1 plants and IH were examined at 72 hpi. The infection process was classified into four stages: penetration phase (PP), biotrophic phase (BP), necrotrophic phase with NH, which are confined within the initially penetrated epidermal cells (NP1), and necrotrophic phase with NH, which spread into the surrounding cells (NP2). Values are expressed as mean from three independent experiments, each containing six biological replicates. The student *t* test was used for statistical analysis, NS., not significant; *, *p*<0.05; **, *p*<0.01.

In this research, using rice-FOX Arabidopsis lines, we identified the rice *eIF2*β (*Os03g0333300*) variant transcript (Os03g0333300-2), which impacts NHR to *P. oryzae* and HR to *C. higginsianum* in the leaves of Arabidopsis plants. The impact of Os03g0333300-2 expression on disease resistance is dependent on leaf age and varies depending on the type of resistance involved in Arabidopsis. In addition to our results, Dubouzet et al. also identified AK072674 (Os03g0333300-2), which conferred resistance to *Pseudomons syringae* pv *tomato DC3000* and *C. higginsianum* in their rice-FOX Arabidopsis lines (Dubouzet et al. 2011). This response to *C. higginsianum* differs from our study, but this would be due to differences in experimental conditions. Nonetheless, these results suggest that overexpression of Os03g0333300-2 could modify defense responses to pathogens in a leaf age-dependent manner in Arabidopsis.

According to the RiceXPro database (https://ricexpro.dna.affrc.go.jp/ (Accessed Jan 29, 2025)), the expression of Os03g0333300-2 is regulated with the fungal pathogen *P. oryzae* in rice. The expression of Os03g0333300-2 is suppressed under compatible and incompatible interactions in leaves (Supplementary Figure S5). Further, the expression is suppressed under incompatible interactions but induced under compatible interactions in roots (Supplementary Figure S6). The expression pattern of Os03g0333300-2 differs from those of Os03g0333300-1 and Os03g0333300-3 (Supplementary Figures S5, S6). This finding suggests that Os03g0333300-2 may play a unique role in regulating rice resistance to *P. oryzae* in rice.

*AtEIF2*β (*At5g20920*) is the closest homologue of *Os03g0333300* in Arabidopsis. Another name for the AtEIF2β is EMBRYO DEFECTIVE 1401, which indicates that the null mutant of *AtEIF2*β gene is embryo-lethal. Therefore, knockout mutants of the *AtEIF2*β gene in Arabidopsis cannot be analyzed. Furthermore, the homolog of the Os03g0333300-2 variant has not been found in Arabidopsis (TAIR: https://www.arabidopsis.org/ (Accessed Jan 29, 2025)). Although it is possible that the variant has not been identified yet in Arabidopsis, it may only exist in rice.

Global changes in translation during plant development and growth have been well-documented (Merchante et al. 2017). Research has shown that translation decreases with age (Kawaguchi et al. 2003; Yamasaki et al. 2015), and that polysome content is higher in actively dividing tissues (Bensen et al. 1988; Mason et al. 1988). More recently, cell division cycle 123 (CDC123) was identified as a crucial activator of protein translation during effector-triggered immunity (ETI) in plants (Chen et al. 2023). Elevated ATP levels during ETI enhance CDC123-mediated assembly of the eIF2 complex (eIF2αβγ), thereby promoting translation. These findings suggest that translation plays a key role in regulating age-dependent resistance responses to pathogens in plants.

Os03g0333300-2 potentially encodes a short protein of 100 amino acids (Supplementary Figures S2, S3). While the function of Os03g0333300-2 in rice remains unclear, it might interfere with the eIF2β function. At the RNA level, Os03g0333300-2 transcripts could potentially interfere with the translation initiation of eIF2β (Os03g0333300-1) transcripts. At the protein level, the short protein might hinder the assembly of the eIF2 complex (eIF2αβγ).

In conclusion, our study showed that overexpression of Os03g0333300-2 reduced NHR to *P. oryzae* and influenced HR to *C. higginsianum* in a leaf age-dependent manner. These findings suggest that the rice *eIF2*β variant Os03g0333300-2 could contribute to defense responses in Arabidopsis, potentially through its involvement in eIF2-dependent translation regulation of resistance responses. Further research is needed to elucidate the precise mechanisms by which the rice *eIF2*β variant influences resistance responses in plants. Understanding these mechanisms could lead to strategies for enhancing plant defense against pathogens, particularly in the context of age-dependent regulation.

## Abbreviations

eIF2β: eukaryotic translation initiation factor 2 beta subunit
FOX: full-length cDNA overexpressing
HR: host resistance
NHR: nonhost resistance

## Description of Supplementary Files

Materials and Methods

Supplementary Figure S1. The information of *Os03g0333300* gene.

Supplementary Figure S2. The nucleotide sequence and deduced amino acid sequence of AK072674 cDNA.

Supplementary Figure S3. Alignment of the amino acid sequences of Arabidopsis EIF2β, rice Os03g0333300-1, Os03g0333300-2, and Os03g0333300-3.

Supplementary Figure S4. Reverse transcription-polymerase chain reaction (RT-PCR) analysis of AK072674 (Os03g0333300-2) expression in transgenic Arabidopsis plants.

Supplementary Figure S5. Expression profiles of the three splice variants (Os03g0333300-1, Os03g0333300-2, and Os03g0333300-3) in whole leaf inoculated with *Pyricularia oryzae.*

Supplementary Figure S6. Expression profiles of the three splice variants (Os03g0333300-1, Os03g0333300-2, and Os03g0333300-3) in whole root inoculated with *Pyricularia oryzae.*
