# Supplementary material for: Overexpression of a rice eIF2β variant modulates leaf age-dependent resistance to pathogens in Arabidopsis thaliana
Source: Plant Biotechnol (Tokyo). 2025 Mar 25;42(1):99–104. doi: 10.5511/plantbiotechnology.24.1206a (PMC12622894; doi:10.5511/plantbiotechnology.24.1206a)
Supplement: Supplementary Data [file plantbiotechnology-42-1-24.1206a-s001.pdf]

Supplementary Files for

Overexpression of a rice *eIF2β* variant modulates leaf age-dependent resistance to pathogens in *Arabidopsis thaliana*

The PDF file includes:

- Materials and Methods
- References
- Figures S1 to S6

Materials and Methods

Plant materials and growth conditions

The plants used in this study included Col-0 (wild-type), *pen2 mol2 pmr5 gl1 NahG* (*pm5gN*) (Shimizu et al. 2021), r1 (original name K19720) (Dubouzet et al. 2011), and #52 (this study), which all on the Col-0 background. Arabidopsis plants were grown in a growth room under controlled conditions as described previously (N. Maeda et al. 2023).

Arabidopsis plants were grown on Murashige and Skoog plates in a growth room for three weeks in short-day conditions (9:15 L:D) at 22 °C (in 100 μmol $m^{-2}sec^{-1}$ fluorescent illumination). Then, the plants were transferred to soil and grown in a growth chamber for 4 weeks, where they continued to grow in short-day conditions (9:15 L:D) at 22 °C (in 100 μmol $m^{-2}sec^{-1}$ fluorescent illumination) and 50% humidity. The plants were watered every three days.

Fungal strains and media

We obtained *Pyricularia oryzae* isolate Hoku 1 (race 007) from H. Koga (Ishikawa Prefectural University). *Colletotrichum higginsianum* (MAFF305635) and *Colletotrichum nymphaeae* (MAFF240037) were obtained from the Ministry of Agriculture, Forestry and Fisheries GenBank, Japan. *P. oryzae* culture was maintained on oatmeal medium at 25 °C in the dark. Cultures of fungal isolates of *Colletotrichum* were maintained on PDA medium at 25 °C in the dark. For inoculation, *P. oryzae* and *C.nymphaeae* were cultured under a 9 h light/15 h dark cycle.

Fungal inoculation

To measure the penetration rates of fungal pathogens, a conidial suspension of each fungus (*P. oryzae*, 5×10^4 conidia/mL; *Colletrichum*, 1×10^5 conidia/mL) was inoculated onto leaves (young leaf, leaf number 13; old leaf, leaf number 8) of rosettes on Arabidopsis (i.e., leaves numbered from oldest to youngest) in the morning (10:00 a.m., am-inoculation) and the evening (5:00 p.m., pm-inoculation). Inoculated plants were maintained in a growth chamber with saturating humidity in short-day conditions (9 h:15 h light:dark) at 22 °C (in 100 μmol $m^{-2}sec^{-1}$ fluorescent illumination). Inoculated leaves were harvested at 72 h post-inoculation (hpi).

To quantify cell penetration for *P. oryze* and *C. higginsianum*, we examined germinated fungal sporelings that had developed appressoria (six leaves from six independent plants per experiment and genotype). We evaluated a minimum of 100 appressoria/leaves. We detected successful penetration of fungal pathogens by observing

autofluorescence or hyphal elongation at infection sites with fluorescence and bright-field microscopy. Each plant genotype was quantified in three independent experiments.

To quantify cell penetration for *C.nymphaeae*, we examined germinated fungal sporelings. We detected successful penetration of *C.nymphaeae* by observing autofluorescence or hyphal elongation at infection sites with fluorescence and bright-field microscopy. Each plant genotype was quantified in three independent experiments.

To quantify fungal growth for *C. higginsianum* in Arabidopsis leaves, we examined inoculated area and measured fungal growth with bright-field microscopy (six leaves from six independent plants per experiment and genotype). Each plant genotype was quantified in three independent experiments.

Rice-FOX Arabidopsis pm5gN lines and P. oryzae screening

The Agrobacterium library of rice full-length cDNAs was obtained from RIKEN. Arabidopsis *pm5gN* plants were transformed using the Agrobacterium library and the transformed *pm5gN* plants expressing rice full-length cDNAs (rice-FOX Arabidopsis *pm5gN* lines) were generated. We inoculated the rice-FOX Arabidopsis *pm5gN* lines with *P. oryzae* by applying 5 μl droplets (5×10^4 spores/mL) of *P. oryzae* onto young leaves (leaf number 13) of rosettes on Arabidopsis (i.e., leaves numbered from oldest to youngest) in the morning (10:00 a.m.). Then, inoculated plants were maintained in a growth chamber with saturating humidity in short-day conditions (9 h:15 h light:dark) at 22 °C (in 100 μmol $m^{-2}sec^{-1}$ fluorescent illumination). Inoculated leaves were harvested at 72 hpi. To quantify cell penetration, we examined germinated fungal sporelings that had developed appressoria. We evaluated a minimum of 100 appressoria/leaves. We detected successful penetration of *P. oryzae* by observing autofluorescence or hyphal elongation at infection sites with fluorescence and bright-field microscopy.

We identified the candidate NHR-related lines with a penetration rate different from the rate of control *pm5gN* plants from a screen of approximately 1,000 rice FOX Arabidopsis *pm5gN* lines. Screening of the candidate NHR-related lines was repeated thrice for verification. For further examination, the selected candidate lines were inoculated with *P. oryzae* at 10:00 a.m. (am-inoculation) and 5:00 p.m. (pm-inoculation) on young and old leaves (young leaf, leaf number 13; old leaf, leaf number 8) of rosettes on Arabidopsis. Each plant was quantified in three independent experiments.

Reverse transcription polymerase chain reaction (*RT-PCR*)

Total RNA was extracted from 3-week-old plants using RNeasy (QIAGEN). The extracted RNA was then used as a template for RT-PCR for AK072674 (Os03g0333300-2) and Actin 2

using the following primers: # AK072674-F, CATCAGAAACCCTAGCAGGAG; # AK072674-R, GCCTATCACTCATCTCAGATG; ACT2-F, GTTGGGATGAACCAGAAGGA; ACT2-R, GAACCACCGATCCAGACACT. Actin 2 (ACT2) was used as the control.

Alignment of amino acid sequences

According to UniProt (https://www.uniprot.org), the predicted amino acid sequence for AK072674 cDNA (Os03g0333300-2), identified as A0A0P0VX34 (listed in Supplementary Figure S1B), begins with leucine (highlighted by the blue line in Supplementary Figure S2). However, for the alignment presented in Supplementary Figure S3, we opted to use an alternative sequence starting with the first methionine (marked by the red line in Supplementary Figure S2).

References

Dubouzet JG, Maeda S, Sugano S, Ohtake M, Hayashi N, Ichikawa T, Kondou Y, Kuroda H, Horii Y, Matsui M, et al. (2011) Screening for resistance against Pseudomonas syringae in rice-FOX Arabidopsis lines identified a putative receptor-like cytoplasmic kinase gene that confers resistance to major bacterial and fungal pathogens in Arabidopsis and rice. *Plant Biotechnology Journal* 9: 466-485

Maeda N, Matsuta F, Noguchi T, Fujii A, Ishida H, Kitagawa Y, Ishikawa A (2023) The Homeodomain-Leucine Zipper Subfamily I Contributes to Leaf Age- and Time-Dependent Resistance to Pathogens in Arabidopsis thaliana. *Int J Mol Sci* 24

Shimizu S, Yamauchi Y, Ishikawa A (2021) Photoperiod Following Inoculation of Arabidopsis with Pyricularia oryzae (syn. Magnaporthe oryzae) Influences on the Plant-Pathogen Interaction. *Int J Mol Sci* 22

A

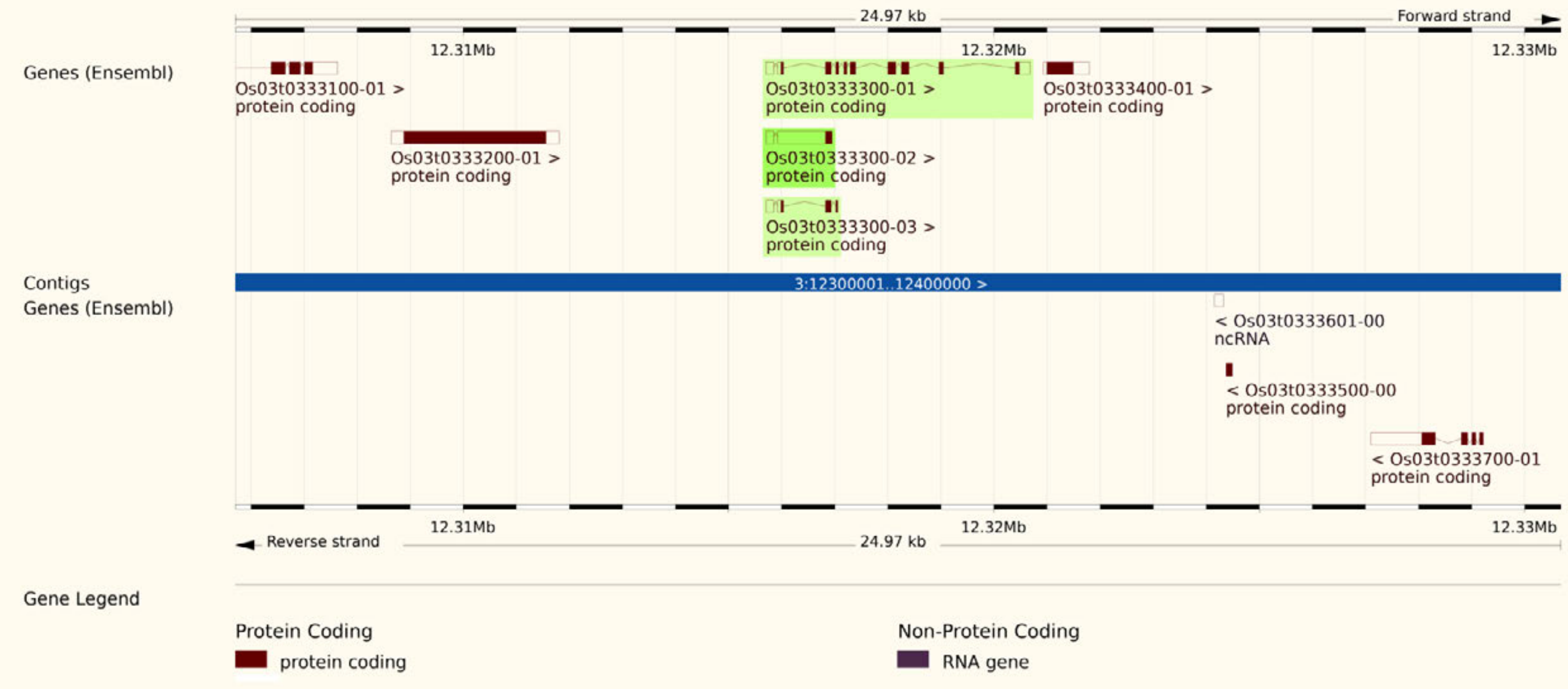

B

Show/hide columns (1 hidden)

Filter

Name	Transcript ID	bp	Protein	Biotype	UniProt	RefSeq	Flags
-	Os03t0333300-01	1220	270aa	Protein coding	A0A8J8Y2P5 Q10LV9	-	Ensembl Canonical
-	Os03t0333300-02	1172	41aa	Protein coding	A0A0P0VX34	-	-
-	Os03t0333300-03	390	62aa	Protein coding	A0A0P0VX12	-	-

Supplementary Figure S1. The information of *Os03g0333300* gene (https://plants.ensembl.org/Oryza_sativa/).
(A) An overview of the information of *Os03g0333300* gene at the gene level. The gene has three transcripts (splice variants: Os03g0333300-1, Os03g0333300-2, and Os03g0333300-3).
(B) The summary of transcripts and their translation lengths of the three splice variants (Os03g0333300-1, Os03g0333300-2, and Os03g0333300-3).

AK072674 (Os03g0333300-2)

```
        10         20         30         40         50         60         70         80         90        100
 Q  G  N  H   P  R  K   R  K  T   E  E  S  K   K  K  D   S  F  S   K  A  *  Q   S  A  H   Q  K  P   *  Q  E  R
caggcaacc acccaagaaa aagaaaaaca gaggagtcga agaaaaagga tagcttcagt aaagcctagc aaagcgccca tcagaaaccc tagcaggagc
       110        120        130        140        150        160        170        180        190        200
  R  R  R   R  R  R   P  P  S  S   S  S  L   S  P  F   Q  V  P  S   P  P  R   R  G  S   S  R  R  V   K  K  A
gccgccgccg ccgccgccgc ccgccgtcgt catcctccct ctcgcccttt caggtcccca gcccgccgcg ccgcggatcg agtcgccgtg tgaagaaggc
       210        220        230        240        250        260        270        280        290        300
  S  V  T   M  A  D  E   E  Q  V   E  R  K   E  E  V  S   E  V  K   S  L  P   F  S  F  F   F  S  F   F  H  L
cagcgtcacc atggcggacg aggagcaggt ggagaggaag gaggaggtct ccgaggtgaa aagcctgccc ttctcgtttt tttttctttt ttttcatttg
       310        320        330        340        350        360        370        380        390        400
 P  C  T  S   D  S  V   R  F  F   G  G  E  G   K  K  R   R  S  D   G  L  G  A   V  P  C   C  A  V   C  V  W  C
ccctgcacat cggattcagt tcggtttttc ggcggggaag ggaagaaacg gcgtagcgat gggctgggtg cggtgccgtg ctgtgctgtc tgtgtttggt
       410        420        430        440        450        460        470        480        490        500
  M  R  D   L  V  G   C  F  G  R   E  S  A   T  M  G   L  F  R  M   F  D  L   C  A  W   I  E  S  V   K  S  V
gtatgcgtga tctggtaggg tgttttgggc gtgaatccgc gaccatgggc ctcttcagga tgtttgatct gtgtgcttgg attgaatcag tgaaatctgt
       510        520        530        540        550        560        570        580        590        600
  R  A  D   *  A  S  E   M  S  G   L  Q  K   *  *  K  K   T  S  E   M  S  D   R  P  D  *   A  M  I   C  F  V
aagagcggat taagcttctg agatgagtgg gcttcaaaaa taataaaaaa aaacatctga gatgagtgat aggccagatt aagcaatgat ttgttttgtt
       610        620        630        640        650        660        670        680        690        700
 H  L  F  V   V  A  C   E  D  *   T  S  *  Q   *  T  P   M  V  I   F  D  G  V   W  T  C   V  *  I   Y  C  Q  C
catcttttg tagtagcttg tgaggactga accagttagc agtgaacacc tatggtgata tttgatggtg tttggacttg tgtctgaata tactgtcagt
       710        720        730        740        750        760        770        780        790        800
  *  P  G   H  Y  Q   C  Y  N  G   P  V  R   I  Y  A   P  Q  C  G   I  F  L   L  I  E   R  M  *  S   C  S  L
gctagcctgg tcattatcag tgttataatg gccctgtcag gatatatgca ccacaatgtg ggatttttct gttgattgaa cggatgtaga gctgtagcct
       810        820        830        840        850        860        870        880        890        900
  *  G  D   Q  Q  I  R   F  I  I   C  V  L   S  M  A  V   T  C  Y   A  *  G   L  S  C  A   T  K  G   *  H  H
gtaaggagat caacaaatta gattcatcat ttgtgttttg agcatggcgg tcacatgtta tgcctaagga ttatcatgtg caacaaaggg ttaacatcac
       910        920        930        940        950        960        970        980        990       1000
 N  L  I  K   S  K  *   N  V  T   P  W  W  I   S  A  F   Y  L  F   V  I  S  L   S  Q  L   F  L  V   S  L  L  Y
aatttgataa aaagcaagtg aaatgttact ccgtggtgga tttcagcatt ttatttgttt gtaatcagcc tttcacaatt atttcttgtc agccttctat
      1010       1020       1030       1040       1050       1060       1070       1080       1090       1100
  H  F  S   L  Y  Q   R  L  V  D   N  V  C   D  V  L   *  L  T  P   F  D  P   T  K  K   K  K  K  K   K  V  V
atcattttc cttgtaccag cggcttgttg acaatgtttg tgatgttttg tagcttactc cttttgaccc aaccaagaaa aagaagaaga agaaggttgt
      1110       1120       1130       1140       1150       1160       1170       1180       1190       1200
  I  Q  D   P  S  D  E   V  D  K   L  A  E   K  T  E  S   L  T  G   I  L  L   M  L
gatccaagat ccatctgatg aggtggataa gttggcagag aaaactgaga gcttgacagg tatactttta atgctgc
```

Supplementary Figure S2. The nucleotide sequence and deduced amino acid sequence of AK072674 cDNA (Os03g0333300-2).
The amino acid sequence of protein A0A0P0VX34, shown in Supplementary Figure S1B, is highlighted in blue. The longest open reading frame (ORF), utilized for the sequence alignment in Supplementary Figure S3, is marked in red.

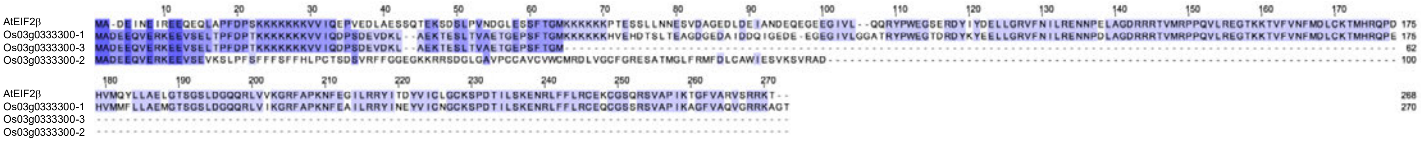

Supplementary Figure S3. Alignment of the amino acid sequences of Arabidopsis EIF2β, rice Os03g0333300-1, Os03g0333300-2, and Os03g0333300-3. Percentage identity is shown by blue colour.

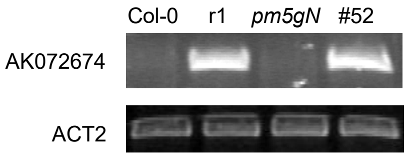

Supplementary Figure S4. Reverse transcription-polymerase chain reaction (RT-PCR) analysis of AK072674 (Os03g0333300-2) expression in transgenic Arabidopsis plants. Actin 2 (ACT2) was used as the control.

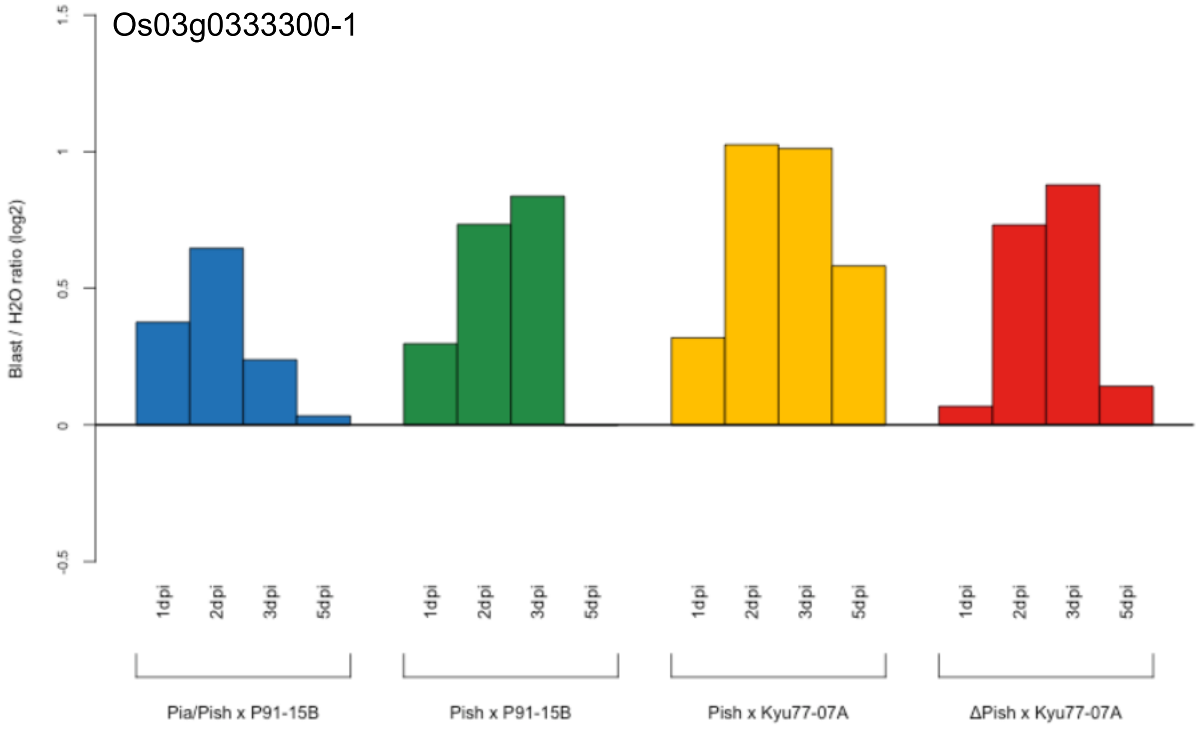
Os03g0333300-1
Blast / H2O ratio (log2)
1.5
1
0.5
0
-0.5
1dpi
2dpi
3dpi
5dpi
Pia/Pish x P91-15B
Pish x P91-15B
Pish x Kyu77-07A
ΔPish x Kyu77-07A

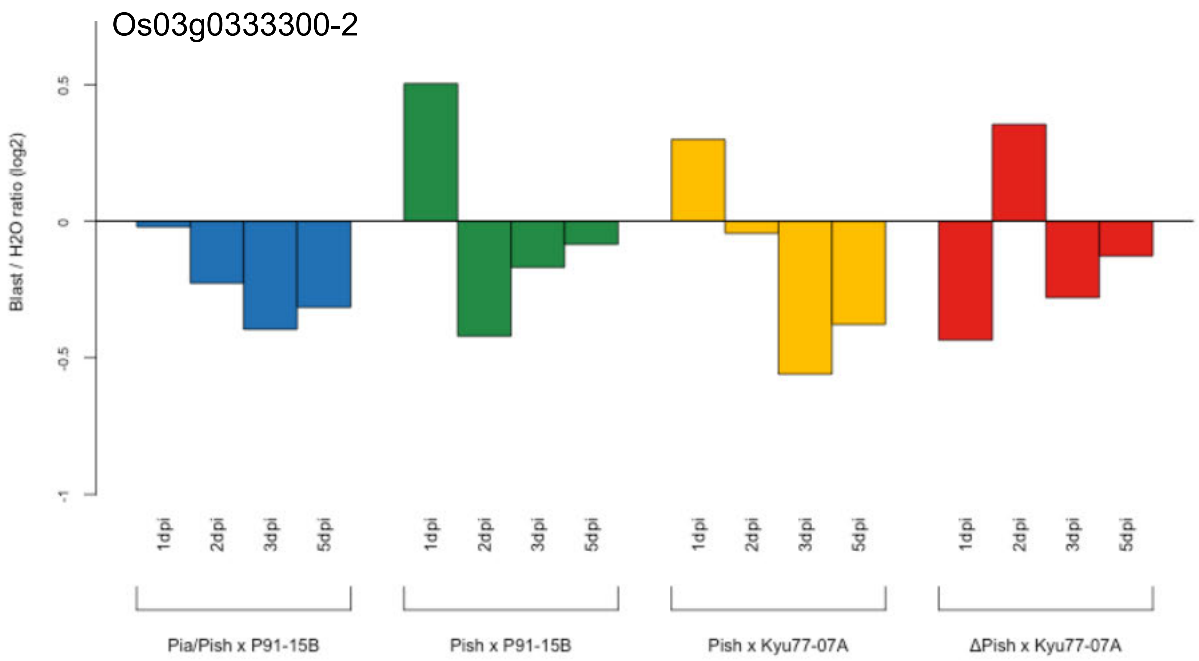
Os03g0333300-2
Blast / H2O ratio (log2)
0.5
0
-0.5
-1
1dpi
2dpi
3dpi
5dpi
Pia/Pish x P91-15B
Pish x P91-15B
Pish x Kyu77-07A
ΔPish x Kyu77-07A

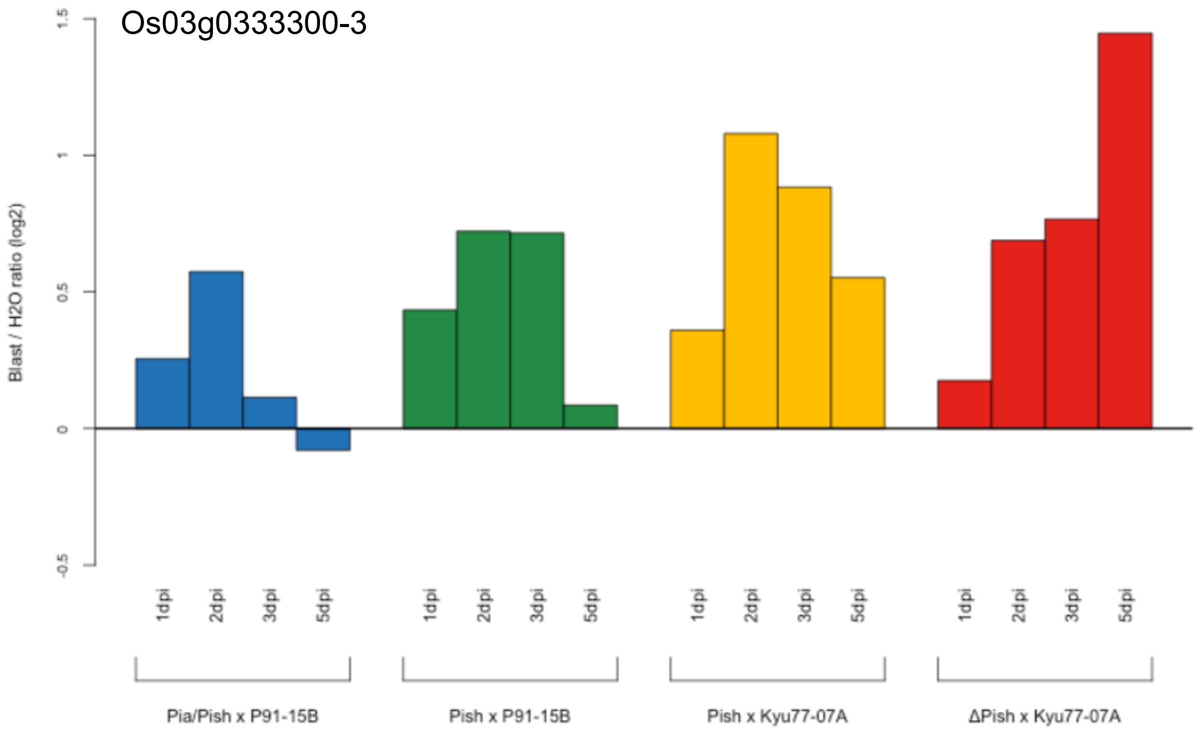
Os03g0333300-3
Blast / H2O ratio (log2)
1.5
1
0.5
0
-0.5
1dpi
2dpi
3dpi
5dpi
Pia/Pish x P91-15B
Pish x P91-15B
Pish x Kyu77-07A
ΔPish x Kyu77-07A

Supplementary Figure S5. Expression profiles of the three splice variants (Os03g0333300-1, Os03g0333300-2, and Os03g0333300-3) in whole leaf inoculated with *Pyricularia oryzae* (syn. *Magnaporthe oryzae*).

Gene expression profile of *Os03g0333300* gene in rice leaves in response to the blast fungus, *Pyricularia oryzae* (https://ricexpro.dna.affrc.go.jp/RXP_3001/index.php). Three lines of rice cultivar Nipponbare carrying the blast resistance genes (*Pia, Pish*) were inoculated with two strains of *P. oryzae* harboring *AVR-Pia* and *AVR-Pish* (https://ricexpro.dna.affrc.go.jp/RXP_3001/details_of_materials.pdf). Rice seedlings at the 4-leaf stage were inoculated with the conidial suspension of *M. oryzae* and the leaves were harvested at 1, 2, 3, and 5 days post inoculation (dpi).

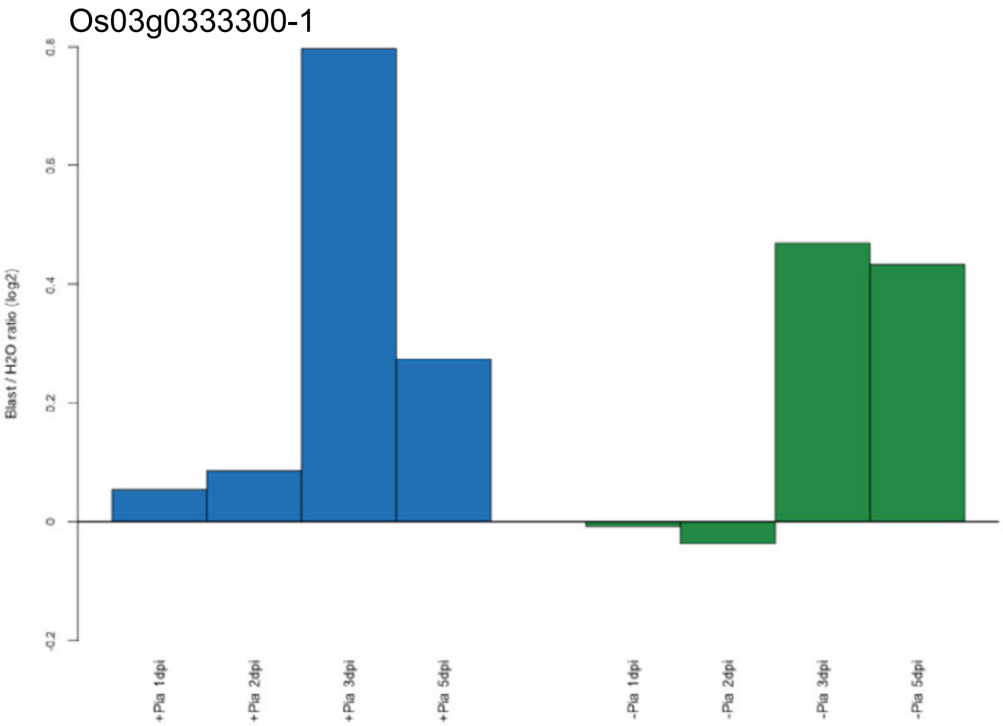
Os03g0333300-1
Blast / H2O ratio (log2)
0.8
0.6
0.4
0.2
0
-0.2
+Pia 1dpi
+Pia 2dpi
+Pia 3dpi
+Pia 5dpi
-Pia 1dpi
-Pia 2dpi
-Pia 3dpi
-Pia 5dpi

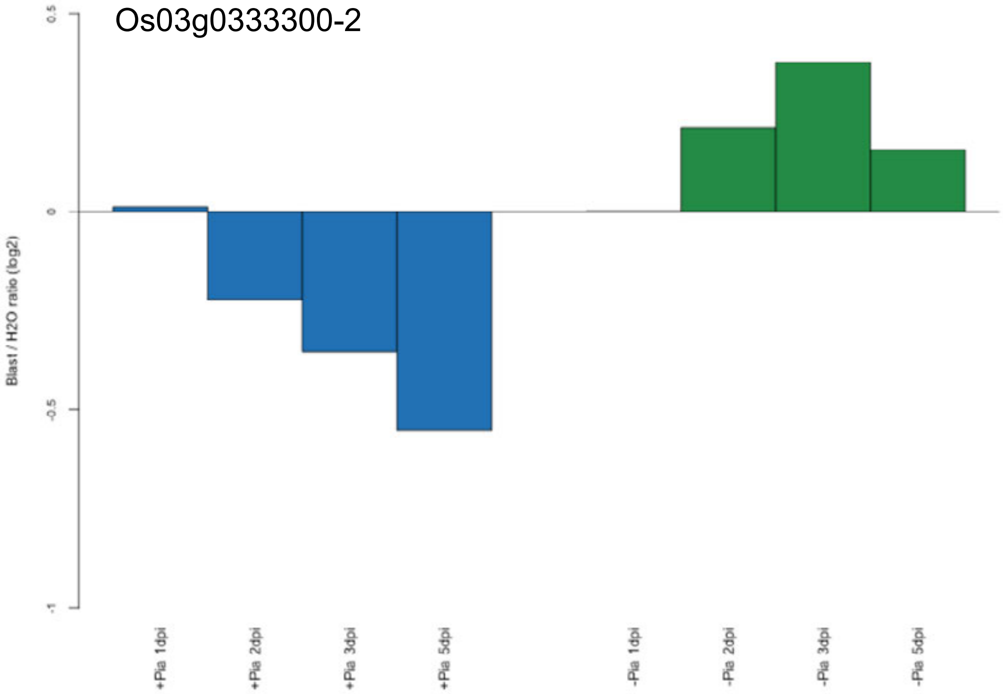
Os03g0333300-2
Blast / H2O ratio (log2)
0.5
0
-0.5
-1
+Pia 1dpi
+Pia 2dpi
+Pia 3dpi
+Pia 5dpi
-Pia 1dpi
-Pia 2dpi
-Pia 3dpi
-Pia 5dpi

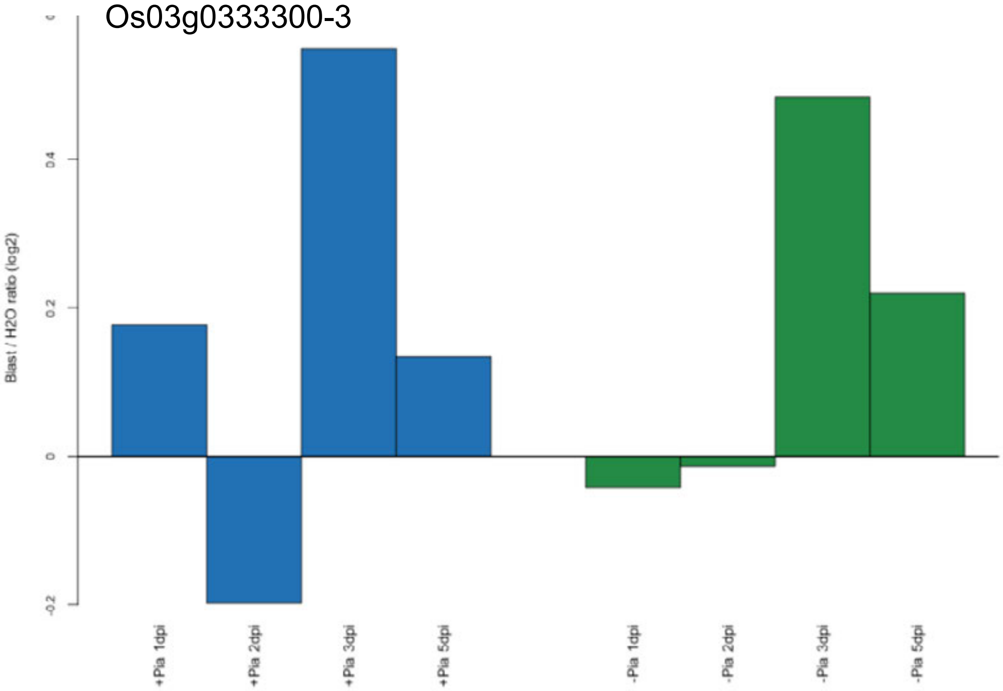
Os03g0333300-3
Blast / H2O ratio (log2)
0.4
0.2
0
-0.2
+Pia 1dpi
+Pia 2dpi
+Pia 3dpi
+Pia 5dpi
-Pia 1dpi
-Pia 2dpi
-Pia 3dpi
-Pia 5dpi

Supplementary Figure S6. Expression profiles of the three splice variants (Os03g0333300-1, Os03g0333300-2, and Os03g0333300-3) in whole root inoculated with *Pyricularia oryzae* (syn. *Magnaporthe oryzae*). Gene expression profile of *Os03g0333300* gene in rice roots in response to the blast fungus, *Pyricularia oryzae* (https://ricexpro.dna.affrc.go.jp/RXP_3003/index.php). Two isogenic rice cultivar Nipponbare, (+*Pia*) and (-*Pia*), were inoculated with strains of *P. oryzae* harboring *AVR-Pia* (https://ricexpro.dna.affrc.go.jp/RXP_3003/Inoculation_methods_20120718.pdf). The roots of rice seedlings were inoculated with the conidial suspension of *M. oryzae* and harvested at 1, 2, 3, and 5 days post inoculation (dpi).
